# Development of a Molecular Marker Based on the Mitochondrial Genome for Detection of *Cyclospora cayetanensis* in Food and Water Samples

**DOI:** 10.3390/microorganisms10091762

**Published:** 2022-08-31

**Authors:** Mauricio Durigan, Emma Patregnani, Gopal R. Gopinath, Laura Ewing-Peeples, Chaeyoon Lee, Helen R. Murphy, Sonia Almeria, Hediye Nese Cinar, Flavia Negrete, Alexandre J. da Silva

**Affiliations:** Office of Applied Research and Safety Assessment, Center for Food Safety and Applied Nutrition (CFSAN), U.S. Food and Drug Administration, Laurel, MD 20708, USA

**Keywords:** *Cyclospora cayetanensis*, detection marker, food safety, environmental samples, agricultural water

## Abstract

*Cyclospora cayetanensis* is a coccidian parasite that causes diarrheal illness outbreaks worldwide. The development of new laboratory methods for detection of *C. cayetanensis* is of critical importance because of the high potential for environmental samples to be contaminated with a myriad of microorganisms, adversely impacting the specificity when testing samples from various sources using a single molecular assay. In this study, a new sequencing-based method was designed targeting a specific fragment of *C. cayetanensis* *cytochrome oxidase gene* and developed as a complementary method to the TaqMan qPCR present in the U.S. FDA BAM Chapter 19b and Chapter 19c. The comparative results between the new PCR protocol and the qPCR for detection of *C. cayetanensis* in food and water samples provided similar results in both matrices with the same seeding level. The target region and primers in the protocol discussed in this study contain sufficient *Cyclospora*-specific sequence fidelity as observed by sequence comparison with other Eimeriidae species. The sequence of the PCR product appears to represent a robust target for identifying *C. cayetanensis* on samples from different sources. Such a sensitive method for detection of *C. cayetanensis* would add to the target repertoire of qPCR-based screening strategies for food and water samples.

## 1. Introduction

*Cyclospora cayetanensis* is a coccidian parasite that causes a human-specific gastrointestinal disease called cyclosporiasis. It has a direct fecal–oral transmission cycle. and the transmission occurs when sporulated oocysts of the parasite are ingested through consumption of contaminated food or water [1,2,3]. After ingestion of oocysts, symptoms of cyclosporiasis begin within an average of 7 days (ranging from 2 days to ≥2 weeks post ingestion). Although the course of the infection can be more severe in immunosuppressed patients, cyclosporiasis is normally self-limiting. Infected individuals shed unsporulated oocysts; once outside the host, the oocysts can sporulate and become infectious within 7–15 days, depending on ideal environmental factors [4,5].

Cyclosporiasis is becoming a significant public health concern in food production. The detection of *Cyclospora cayetanensis* in produce should be considered a possible risk to public health [6]. Oocysts of this parasite have already been detected in fresh produce items such as lettuce, parsley, green onion, cucumber, celery, tomato, spinach, basil, blueberries, and raspberries, among others, in many surveillance studies worldwide [2,7,8,9,10]. In 2018, *C. cayetanensis* was identified for the first time in produce grown in the United States (“Statement from FDA Commissioner Scott Gottlieb, M.D., on the FDA’s ongoing efforts to prevent foodborne outbreaks of *Cyclospora.* Available online: https://www.fda.gov/news-events/press-announcements/statement-fda-commissioner-scott-gottlieb-md-fdas-ongoing-efforts-prevent-foodborne-outbreaks (accessed on 28 August 2022)”. Oocysts of *C. cayetanensis* have also been identified in farm workers and food handlers, which reinforces the need for the development, implementation, and monitoring of on-farm control measures in endemic areas [4,11].

Over the last few years, outbreaks and sporadic cases of cyclosporiasis associated with the consumption of fresh produce were reported in Latin America [4], Europe [12,13], and North America [12,14,15]. In the United States, where *C. cayetanensis* is reportable in 43 states, the District of Columbia, and New York City [15], foodborne outbreaks of cyclosporiasis have been reported annually after the initial reporting in 1996. Since 2013, there has been a continuous increase in reported domestic cases (i.e., when there is no travel related to the 14-day period before illness onset) and multistate outbreaks. In 2018 and 2019, the number of cases, hospitalizations, and the number of states reporting cyclosporiasis cases increased significantly (“Outbreak Investigations and Updates. Available online: https://www.cdc.gov/parasites/cyclosporiasis/outbreaks/index.html (accessed on 28 August 2022)”. 

Waterborne infectious diseases remain a major source of morbidity and mortality in the world, and parasitic protozoan outbreaks are one of the leading causes of 1.7 billion cases of diarrhea [16]. Most of these protozoa are capable of infecting humans through the fecal–oral route in which land and rivers are contaminated by feces of both human and animal origin [17,18]. Outbreaks of waterborne protozoa parasites have been identified in North America [19,20,21] and worldwide [16]. In this context, agricultural water may serve as a vehicle for contamination of fresh produce during the irrigation process [4,21], posing a potentially serious threat to millions of people in the world [17]. *Cyclospora* spp. oocysts have been already detected in several water sources including, rivers, irrigation ponds, wastewater, sewage [22,23,24,25], and even water intended for human consumption [22,26].

The development of new laboratory methods for detection of *C. cayetanensis* is of critical importance. In this context, the Food and Drug Administration (FDA) has developed and validated methods for detection of *C. cayetanensis* in produce and agricultural water (Bacteriological Analytical Manual (BAM) chapter 19b,c). A method first validated by the FDA for the detection of *C. cayetanensis* in cilantro and raspberries [27,28] was later extended for detection in other matrices such as shredded carrots, basil, blackberry, shredded cabbage, romaine lettuce, and parsley [2,27]. More recently, the FDA validated a method for detection of *C. cayetanensis* in agricultural water based on dead-end ultrafiltration [25], published in the FDA’s Bacteriological Analytical Manual (BAM) in chapter 19c [29]. In both methods, the detection of *C. cayetanensis* relies on a specific real-time PCR (qPCR), which targets the *C. cayetanensis* 18S rRNA gene. In a different approach, a molecular marker using the ITS-1 region as a target was developed as an alternative for use in the analyses of berry samples and other fresh produce for *Cyclospora* contamination [30].

Advances in genome sequencing have benefited parasitology in many ways. In the last 7 years, there has been a significant increase in the number of *Cyclospora* sequences in genetic databases covering both whole genome and organellar genome sequences [31,32,33,34,35,36,37]. The development of novel detection and genotyping tools is necessary to optimize, analyze and understand the significance of data collected. The development of highly sensitive and specific detection methods will help to better understand the environmental dissemination dynamics of *C. cayetanensis* oocysts and how contaminated water and fresh produce (including farm workers and food handlers) can affect the food chain and potentially cause waterborne protozoan outbreaks [25].

In this study, we developed and evaluated a molecular marker for detection of *C. cayetanensis* in food and water samples. A new PCR-sequencing-based method named mit3PCR was developed targeting a fragment of the *C. cayetanensis cytochrome oxidase gene* and was developed as a complementary method to the real-time PCR validated by the U.S. FDA BAM Chapters 19b and 19c. The methods described in this study were also used for detection of *C. cayetanensis* in real field samples, i.e., samples collected in the environment and not spiked in the laboratory.

## 2. Materials and Methods

### 2.1. C. cayetanensis Oocysts Isolation and Sample Preparation

The oocysts used in the experiments designed to evaluate this new method were purified from individual human stool samples stored in 2.5% potassium dichromate, as described elsewhere [28]. The study was approved by the Institutional Review Board of the FDA (protocol number 15-039F). The purified oocysts were enumerated using a hemocytometer on an Olympus BX51 microscope (Optical Elements Corporation, Dulles, VA, USA). To avoid pipetting errors, the oocysts were diluted in 0.85% NaCl to reach the desired concentration of 10 oocysts/μL (higher seeding levels) and 6 oocysts/10μL (lower seeding levels) for seeding experiments in water and 10 oocysts/μL (higher seeding levels) and 1 oocyst/μL (lower seeding levels) for seeding experiments in produce. DNA extraction was performed using the FastDNA SPIN Kit for Soil in conjunction with a FastPrep-24 Instrument (MP Biomedicals, Santa Ana, CA) following the procedure described in the FDA’s BAM Chapter 19b [27] for food samples and the procedure described in the FDA’s BAM Chapter 19c [29] for water samples. For water samples only, after the DNA extraction procedure, the DNA extracts were purified further using the QIAquick^®^ PCR Purification Kit as described in the kit’s protocol. Final elution was performed with 30 µL of elution buffer (10 mM Tris·Cl, pH 8.5) to improve DNA recovery as recommended. The DNA samples were stored at 4 °C for up to 5 days or at −20 or −80 °C for longer-term storage. 

### 2.2. Conventional PCR Assay Developed Based on the Mitochondrial Genome

**Figure 1:**
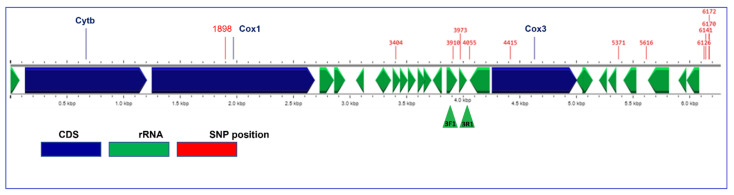
Allelic hotspots and gene annotations of *C. cayetanensis* mitochondrial genome. Annotations of KP231180, the reference mitochondrial genome of *C. cayetanensis* [31], are illustrated. Two coding sequences CDS *cox1* and *cox3* (blue) contain a few polymorphic alleles, but the majority of the allelic hotspots (base-positions marked in red) are distributed across the genome, including among rRNA genes (green) and intergenic regions. The hotspots were identified by manually curating multiple alignments of genomes with the reference genome KP231180 [34,36]. The rRNA-containing segment between 3900 and 4100 with two SNPs was targeted by the mit3PCR developed in this study (green triangles).

The *C. cayetanensis* PCR detection assay reported in this study was developed as a complementary method to confirm qPCR-positive samples. PCR primers were designed to amplify different regions of the *C. cayetanensis* mitochondrial genome based on the GenBank entry KP231180 and available mitochondrial genome sequences [31,35,36]. Each primer pair was tested to select the primer combination with the highest sensitivity and specificity followed by DNA sequencing analysis for detection of *C. cayetanensis*. Initial set of primers tested included those specifically designed in a conserved region and near known allelic hotspots on *C. cayetanensis* mitochondria [36] (Figure 1). The complete list of primers designed for this study is presented in Supplementary File S1. Tm values were calculated according to “Melting Temperature (Tm) Calculation” tool [38]. Annealing temperatures were tested initially at 5 °C below the Tm of the primers and then increased to improve the stringency of the test. Different conditions were tested for optimization regarding the concentration of primers, MgCl, and the inclusion of non-fat dried milk solution. The combinations of primers designed to detect *C. cayetanensis* are presented in Supplementary File S2.

Primers were designed using Primer 3 Plus [39]. DNAStar Lasergene version 17.3 (DNAStar, Madison, WI, USA) was used to identify for elimination undesired complementary sequences and hairpins. For a PCR positive control, a DNA target was commercially synthesized as a 100-bp ultramer DNA oligo (Integrated DNA Technologies, Inc., Coralville, USA). The selected primer pair targeting a *C. cayetanensis* mitochondrial gene and the synthetic positive control used in this study are listed in Table 1.

PCR reactions were optimized to be performed in a 50µL PCR reaction volume containing 10 µM of each primer, 25 µL AmpliTaq Gold^®^ 360 Master Mix (ABI/Thermo Fisher Scientific, Waltham, MA, USA), 2.5 µL of 2% non-fat dried milk solution, and 1 µL of 25 mM MgCl, to which 5 microliters of DNA template were added. Reactions were run on a Veriti™ thermal cycler with the following cycling conditions: 95 °C, 10 min for initial denaturation; then 35 cycles of 95 °C for 30 s, 54 °C for 30 s, and 72 °C for 1 min; and a final extension at 72 °C for 10 min. PCR products were visualized by agarose gel electrophoresis on a 1.5% agarose gel, stained with ethidium bromide, and visualized using the GBox Chemi XT Imaging system with GeneSnap software (Syngene, Cambridge, UK).

Downstream sequencing analysis was performed on amplicons to verify the specificity of the amplification. The amplicons were purified using the QIAquick purification kit (Qiagen, Germantown, MD, USA) following the manufacturer’s instructions. The purified amplicons were sequenced on both strands (Psomagen Inc., Rockville, MD, USA), and the sequencing data were edited, assembled, and analyzed using SeqMan Pro 14 from DNAStar (Madison, WI, USA).

### 2.3. Specificity Evaluation of Conventional mit3PCR Assay

NCBI nucleotide BLAST suite was used to confirm the specificity of the proposed assay for the detection of *C. cayetanensis* regarding nucleotide sequences. Mitochondrial genome sequences for the Reference Genome KP231180 and other *Cyclospora* mitochondrial assemblies were obtained from the CycloTrakr database (BioProject: PRJNA357477) under the GenomeTrakr project on NCBI. Seventy-six mitochondrial sequences from member species of the Eimeriidae family (Supplementary File S3) were obtained from NCBI GenBank. The illustration for Figure 1 was carried out on the ProkSee webserver, an expert system for genome assembly and annotation at beta.proksee.ca. Multiple alignments of sequences to identify taxon-specific allelic profile were carried out using MAFFT (MAFFT. Available online: https://www.ebi.ac.uk/Tools/msa/mafft/ (accessed on 28 August 2022) and Geneious Prime 12 suite. For this, about 200 bases spanning the target region and flanking sequences were first manually generated from each of the genome assemblies.

Specificity was also evaluated in vitro by using an exclusivity panel consisting of DNA samples from foodborne bacterial and parasitic pathogens in addition to in silico testing using sequences available in GenBank. This panel included DNA from the following microorganisms: *Cryptosporidium parvum*, *Cryptosporidium hominis*, *Cyclospora papionis*, *Eimeria acervulina*, *Eimeria tenella*, *Eimeria maxima*, *Entamoeba histolytica*, *Giardia duodenalis*, *Blastocystis hominis*, *Plasmodium falciparum*, *Neospora caninum*, *Toxoplasma gondii*, *Salmonella sp.*, *Escherichia coli*, and *Trypanosoma cruzi*. *Cyclospora cayetanensis* DNA was used as a positive control. Since the proposed method is a sequencing-based method, if any of the tested DNA generated any band in the gel, the fragment would be submitted for Sanger sequencing.

### 2.4. Evaluation of Sensitivity and Comparison of the New Conventional PCR Assay with Validated qPCR for Food and Water Samples

#### 2.4.1. Seeded Food Samples

The mit3PCR assay was compared with the BAM Chapter 19b real-time PCR for sensitivity and performance using a set of DNA samples extracted from food samples. A total of 18 cilantro and 18 raspberry samples seeded at different concentrations of *C. cayetanensis* oocysts were prepared. The samples were seeded with a suspension of *C. cayetanensis* oocysts originating from an Indonesian patient from which the mitochondrial genome sequence was known. The 25 g of cilantro and 50 g of raspberries were seeded with 5 (n = 4), 10 (n = 4), 20 (n = 3) and 200 (n = 3) oocysts of *C. cayetanensis*. Four unseeded controls were added to each different produce. DNA was extracted from these samples, and Real-Time qPCR was performed on an Applied Biosystems 7500 Fast Real-Time PCR System (ThermoFisher Scientific, Waltham, MA, USA) in fast mode using the QuantiFast Multiplex PCR +R Kit (Qiagen, Hilden, Germany). The procedures for washing the samples, DNA extraction, and qPCR were performed according to the BAM Chapter 19b [27].

#### 2.4.2. Seeded Agricultural Water Samples

The mit3PCR assay was also compared with the BAM Chapter 19c real-time PCR regarding sensitivity and performance using a set of 30 agricultural water samples previously analyzed according to the BAM Chapter 19c [29], in which a set of agricultural water samples was seeded with different concentrations of oocysts in 10 L water samples, i.e., 200 (n = 6), 100 (n = 3), 25 (n = 6), 12 (n = 3), and 6 (n = 12), oocysts. Unseeded water samples from the same source (n = 12) were processed with seeded samples to serve as negative controls. All samples were filtered using the dead-end ultrafiltration (DEUF) technique and processed for recovery and concentration of oocysts, followed by DNA extraction and qPCR according to the BAM Chapter 19c. The DNA extracts, previously evaluated for qPCR, were then analyzed for the mit3PCR in all seeding levels to compare the detection limits of both techniques.

#### 2.4.3. Detection in Surface Water Samples

The mit3PCR assay was also tested in surface water samples previously collected from the Chesapeake and Ohio Canal, abbreviated as the C&O Canal at Lock 22 (39°03′13.1″ N, 77°17′20.0″ W) [25]. The DNA extracts from these samples were tested and compared with the qPCR from the BAM Chapter 19c to demonstrate the usefulness of this method in real field samples.

### 2.5. Statistical Analysis of qPCR and Conventional PCR Detection Rates in Seeded Produce Samples and Agricultural Water

Two-tailed *p* values were calculated with Fisher’s exact test using the software RStudio (R^®^ statistical software v.3.3.0. R studio team 2015, Boston, USA) to identify significant differences in detection rates between detection methods (mit3PCR or qPCR) for the different seeding levels, produce matrices, and water. *p* ≥ 0.05 indicates no significant difference.

## 3. Results

### 3.1. Evaluation of Molecular Markers and Specificity Analysis

In the first round of the evaluation of the designed molecular markers, different reactions were evaluated regarding the expected fragment size and specificity of *C. cayetanensis* (Supplemental Files S1 and S2). Some of the amplified products covered known allelic hotspots [36], illustrated in Figure 1. These included the proline/serine-rich (SP-rich) regions between 3300 and 4200 in rRNA loci that interact with other proteins as previously described [34] and were further used to complement epidemiological case linkages in their study [37]. The tandem-repeat terminal region between 6100 and 6200 with characterized InDels and SNPs [34,40] was also evaluated with other targets spanning KP231180. The results obtained with primers 3F1 and 3R1 spanning the genomic segment with rRNA gene loci (Figure 1) were considered the most satisfactory. It provided a well-defined band in the gel of the expected fragment size (182 bp), Sanger sequencing yielded expected target sequence, and no cross-amplification was observed with any of the protozoan parasite DNA samples from the exclusivity panel. Other fragments obtained from the different combinations of primers presented less efficiency to reproduce PCR results and less accuracy when submitted to Sanger sequencing. The forward primer 3F1 was designed based on positions 3832–3858, and the reverse primer 3R1 was designed based on positions 3992–4013 of the reference genome GenBank entry KP231180. This conventional PCR assay was selected among tested markers for the detection of *C. cayetanensis* and named mit3PCR.

**Figure 2:**
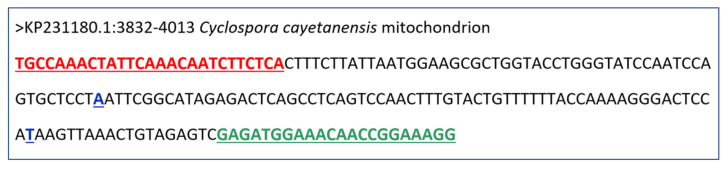
Annotations of the product of mit3PCR by 3F1–3R1 primers. 3F1 and 3R1 primers generated 182 bp product spanning 3832 to 4013 bases inclusive on the reference mitochondria genome KP231180. The forward (red) and reverse (green) primers were also used for sequencing the PCR amplicons. There are two known allelic hotspots (blue) [36] at 3910 (at A to C) and 3973 (T to G) that have been reported to be highly discriminatory with various sample collections (HNC, GG, and AJD personal communication).

The 182-bp amplicon representing a segment 3832–4013 (Figure 2) was used to query known mitochondria sequences from the member species of the Eimeriidae family. Multiple genera including *Eimeria*, *Isospora,* and *Lankesteralla* showed homologous sequences to the mitochondrion segment from the *Cyclospora cayetanensis* reference genome KP231180 (Supplementary File S3). Sequences were aligned using MAFFT software to understand the diversity in these homologous sequences and to identify specific variant bases in the primers used for mit3PCR. Many segments of the predicted amplicon sequence were conserved in these species, and specific differences highlighting the heterogeneity in these organisms were also identified. An alignment showing a representative subset of this dataset is provided in Supplementary File S4. Both insertion-deletions (InDels) and allelic differences were observed in this segment across different species using MAFFT multiple sequence alignment and confirmed the specificity of the mit3PCR protocol in detecting and sequencing *Cyclospora* targets.

### 3.2. Evaluation of the New Optimized PCR Assay in Food and Agricultural Water Samples Seeded with Different Numbers of Oocysts

#### 3.2.1. Detection of *C. cayetanensis* in Seeded Food Samples

The comparative results between mit3PCR and the qPCR for the detection of *C. cayetanensis* in food samples are presented in Table 2. The detection limit of mit3PCR was similar for both cilantro and raspberry seeded samples with the fractional results (i.e., in which the low-level seeding resulted in 50% ± 25% positive results according to the FDA Food Program’s “Guidelines for the Validation of Analytical Methods for the Detection of Microbial Pathogens in Foods and Feeds. Available online: https://www.fda.gov/media/83812/download (accessed on 28 August 2022) being obtained with samples seeded with five oocysts. The number of positive samples for *C. cayetanensis* by the mit3PCR was slightly lower on cilantro seeded with five oocysts and raspberries seeded with five and ten oocysts when compared with the validated method [20]. Nevertheless, the detection limit obtained in the fractional results for both qPCR and the mit3PCR were at seeding levels of 5–10 oocysts. Tested samples were positive in all replicates in the high seeding levels (20 and 200 oocysts). No unseeded samples were positive by either method. The sequences obtained from the 182 bp amplicons generated by the mit3PCR were 100% similar to the *C. cayetanensis* sequences from the original oocysts used to seed the samples.

#### 3.2.2. Detection of *C. cayetanensis* in Seeded Agricultural Water Samples

The experiment conducted to detect *C. cayetanensis* in agricultural water samples using the DEUF method with the mit3PCR provided positive results in all 10 L agricultural water samples seeded with 25, 100 and 200 oocysts. Positive rates decreased at seeding levels of 12 (75%) and 6 oocysts (58.3%). Table 3 presents a summary of the results of experiments conducted to compare the detection sensitivity for *C. cayetanensis* in agricultural water samples using BAM 19c and mit3PCR. The sequences obtained from the 182 bp amplicons generated by the mit3PCR were 100% similar to the *C. cayetanensis* sequences from the original oocysts used to seed the samples.

#### 3.2.3. Detection of *C. cayetanensis* in Environmental Water Samples

A total of six samples collected at the C&O canal were analyzed with the mit3PCR developed in this study. These samples had been previously analyzed by the BAM’s validated qPCR method for detection of *C. cayetanensis* in agricultural water [25]. Table 4 presents a summary of the detection of *C. cayetanensis* in the collected environmental samples in which 50% were considered positive using both qPCR, which targets the 18SrRNA gene, and the mit3PCR, which targets the *C. cayetanensis* mitochondrial genome. The DNA sequencing analysis of the 182 bp amplicon obtained with mit3PCR produced sequences that were 100% similar to other *C. cayetanensis* mitochondrial genome sequences within the same positions. Sequences generated in this study have been deposited in GenBank under accession numbers OL375674 and OL375675.

Amplicons generated by the mit3PCR were sequenced, and a 182 base long fragment spanning from 3832 to 4013 base positions on the Reference Genome KP231180 was identified. BLAST analysis of two representative sequences W33 and W43 against GenBank sequences for apicomplexans had matching hits with 100% identity with many *C. cayetanensis* identical assemblies and with 99.45% and 98.9% with other assemblies that presented one and two SNPs different than the sequenced samples, respectively.

## 4. Discussion

In this study, molecular markers were developed based on selected regions of the *C. cayetanensis* mitochondrial genome which resulted in a sensitive detection marker for this protozoan parasite. This assay compared favorably with the FDA-validated qPCR methods for produce [27] and water [29], and the generated amplicons were sequenced and matched 100% of the *C. cayetanensis* sequences in GenBank. The method described in this study was also applied for the detection of *C. cayetanensis* in field samples to serve as a complementary sequence-based detection tool that will support the findings obtained with the FDA qPCR used in the BAM chapter 19b or 19c methods. An ancillary assay targeting a longer fragment that is readily sequenced and genetically distinct could be employed for confirmation of the samples screened as positive by the BAM method [6].

The development of new detection methods is essential to better understand the dispersion of the parasite in the environment and to support outbreak investigations. The BAM Chapter 19b method was used [27,41] for the identification of the parasite in domestically grown produce and supported the epidemiologic evidence that domestically grown salad mix was the source of one of the 2018 outbreaks “Multistate Outbreak of Cyclosporiasis Linked to Fresh Express Salad Mix Sold at McDonald’s Restaurants—United States, 2018: Final Update. Available online: https://www.cdc.gov/parasites/cyclosporiasis/outbreaks/2018/b-071318/index.html (accessed on 28 August 2022)”. 

The mit3PCR-sequencing-based method was developed because of the high potential for environmental water samples to be contaminated with a myriad of microorganisms that may not be represented in genomic databases and might adversely impact the specificity when testing samples from various sources using a single molecular assay. Moreover, DNA sequencing analysis of amplicons is a standard approach in environmental sample testing to further characterize parasites and complement PCR-based detection [23,42,43]. It is an option for providing confidence regarding positive results [30]. Nevertheless, when compared to the validated method for the detection of *C. cayetanensis* based on qPCR, a method based on conventional PCR and Sanger sequencing is more time-consuming [30] and requires more equipment for downstream analysis. Although both methods presented in the current study were able to identify the parasite at the same seeding level, the detection rates may vary among them, which reinforces the need for using the proposed new method to serve as a complementary detection tool. The proposed method could also serve as a complementary detection tool for other methods, such as the qPCR detection method based on the ITS-1 region developed for the detection of *C. cayetanensis* in berry samples and other fresh produce [30].

The 182 bp region and primers in the mit3PCR protocol discussed in this study contain sufficient *Cyclospora*-specific sequence fidelity as observed by sequence comparison with other Eimeriidae species (Supplementary Files S3 and S4). The sequences of any amplicons generated due to cross-reaction with background taxa in mixed samples could be used to resolve the specificity by comparison with a database of mitochondria genomes, as shown in our study. Multiple alignment using MAFFT and Geneious enunciated the ability of the mit3PCR to confirm the presence of *C. cayetanensis* DNA. Amplicon sequences from *Eimeria* and other Eimeriidae species with alleles and InDels characteristic of non-*C. cayetanensis* sequences need to be made available on the CycloTrakr database to expand the efficiency of future analysis. The significant amplification success rates of the developed mitochondrial marker were expected due to the high proportion of mitochondrial genome copies per cell [44].

Recently, it was demonstrated, for the first time, that the mitochondria can be used (in the indel region, marker 8) for genotyping *C. cayetanensis* [40]. Thereafter, mitochondrial markers have been included in genotyping approaches [37,40,45,46,47]. In particular, the alleles identified in mit3PCR are part of a set of alleles reported to be discriminatory in a genotyping scheme for application in clinical samples [37,46]. At the same time, the efficacy of using organellar genomes for source tracking in food safety efforts by state and health public health agencies has to be studied extensively. The sequence of mit3PCR product appears to represent a robust target for identifying *C. cayetanensis* on samples from different sources. This study also adds an efficient PCR strategy to environmental surveillance studies aimed at understanding taxonomic diversity of coccidian species having a high probability of occupying the same environmental sample sources as *Cyclospora* oocysts.

The statistical analysis performed in the comparison between the mit3PCR with the validated qPCR based on the 18SrRNA showed no significant differences between methods regarding detection rates in the different seeding levels for both produce and agricultural water methods. The mit3PCR was also able to provide the same level of detection in the low seeding levels of *C. cayetanensis* in produce [28,48] and agricultural water [25] according to the BAM chapters 19b and 19c [27,29].

To evaluate the robustness of the new detection marker in real environmental samples, DNA samples obtained from surface water sampled at different locations of the C&O canal in Maryland were subjected to the new mit3 PCR. All the samples that previously tested positive by the validated qPCR for *C. cayetanensis* [25] generated the desired amplicon using the mit3PCR. The results we obtained are in convergence with other studies that detected *C. cayetanensis* in environmental water samples [23,24,26,49,50]. However, our results are preliminary and based on a small number of samples, and therefore, they cannot be correlated with the dispersion of *C. cayetanensis* in the environment.

In conclusion, a laboratory PCR method based on the FDA Mitochondrial Reference Genome capable of detecting *C. cayetanensis* in food and water samples was developed in this study. Such a sensitive method for detection of *C. cayetanensis* from environmental samples would add to the target repertoire of qPCR-based screening strategies currently using only the 18SrRNA gene sequences for food and water samples.

## Figures and Tables

**Table 1:** Sequences of PCR primers and positive control used in this study.

Designation	Target	Sequence (5′–3′)	Amplicon Size (bp)
3F1	*C. cayetanensis*	TGCCAAACTATTCAAACAATCTTCTCA	182
3R1		CCTTTCCGGTTGTTTCCATCTC	
*C. cayetanensis* synthetic target		TGCCAAACTATTCAAACAATCTTCATCCAGTGCTCCTATTTTTACCAAAAGGGACTCCATAAGTTAAACTGTAGAGTCGAGATGGAAACAACCGGAAAGG	100

**Table 2:** Detection rates of *C. cayetanensis* in food samples by real-time PCR and conventional PCR assays.

Matrix	Number of Oocysts Seeded	Number of Seeded Samples Tested	No. of Samples Positive with mit3PCR	No. of Samples Positive by BAM qPCR:	*p* ^b^
			(%)	(%)	
Cilantro (25 g) ^a^	0	3	0 (0%)	0 (0%)	1
	5	4	2 (50%)	3 (75%)	1
	10	4	4 (100%)	4 (100%)	1
	20	4	4 (100%)	4 (100%)	1
	200	3	3 (100%)	3 (100%)	1
Raspberries (50 g) ^a^	0	3	0 (0)	0 (0)	1
	5	4	2 (50%)	3 (75%)	1
	10	4	2 (50 %)	3 (75%)	1
	20	4	4 (100%)	4 (100%)	1
	200	3	3 (100%)	3 (100%)	1

^a^ Amount of produce in each sample was designed based on BAM Chapter 19b; ^b^ *p* values calculated using the Fisher’s exact test to compare nested and real-time PCR detection rates. Differences are considered nonsignificant at *p* ≥ 0.05.

**Table 3:** Summary of detection of *C. cayetanensis* in seeded and unseeded agricultural water using DEUF combined with qPCR and mit3PCR.

Matrix	Seeding Level ^a^	No. of Samples Analyzed	No. of Oocysts (L)	qPCR		mit3PCR		*p* ^b^
				No. of Positive Samples	Positive (%)	No. of Positive Samples	Positive (%)	
Irrigation Water (10 L)	0	12	0	0	0%	0	0%	1
	6	12	0.6	8	66.60%	7	58.30%	1
	12	3	1.2	3	100%	2	75%	1
	25	6	2.5	6	100%	6	100%	1
	100	3	10	3	100%	3	100%	1
	200	6	20	6	100%	6	100%	1

^a^ Oocysts were seeded in 10 L agricultural water samples as described in the ‘‘Materials and Methods’’ section; ^b^ *p* values calculated using the Fisher’s exact test to compare nested and real-time PCR detection rates. Differences are considered nonsignificant at *p* ≥ 0.05.

**Table 4:** Summary of detection of *C. cayetanensis* in environmental water samples combined with qPCR and mit3PCR.

Sample	Origin	Turbidity (NTU)	qPCR Result	qPCR Ct ^C^	mit3PCR	Sequencing Result
W33	C&O Canal	10.2	Positive	35.7	Positive	*C. cayetanensis*
W37	C&O Canal	17.3	Negative	Und	Negative	NA
W40	C&O Canal	14.7	Negative	Und	Negative	NA
W41	C&O Canal	14.9	Negative	Und	Negative	NA
W42	C&O Canal	14.6	Positive	36.6	Positive	*C. cayetanensis* **
W43	C&O Canal	16.5	Positive	33.9 ± 0.4	Positive	*C. cayetanensis*
Stool 28 Control ^b,d^	US	N/A	Positive	29 ± 0.2	Positive	*C. cayetanensis*
Stool 19 Control ^b,d^	US	N/A	Positive	27.1 ± 0.3	Positive	*C. cayetanensis*
Oocysts Control ^b,e^	Purified oocysts	N/A	Positive	31 ± 0.3	Positive	*C. cayetanensis*

Table adapted from Durigan et al., 2020 [25] with new data added. ^b^ DNA extracted from clinical samples positive for *C. cayetanensis* and purified oocysts were included for comparison. ^C^ No SD is shown when only one replicate produced a positive result. ^d^ Stools 19 and 28 represent DNA from clinical samples from two individuals positive for *C. cayetanensis* by microscopic and molecular analysis. ^e^ Oocysts control represents DNA from oocysts purified from stool from an Indonesian patient. ** The complete fragment (182 bp) was not obtained.

## Data Availability

The data presented in this study are openly available in GenBank under access numbers OL375674 and OL375675.

## Supplementary Materials

The following supporting information can be downloaded at: https://www.mdpi.com/article/10.3390/microorganisms10091762/s1, Supplementary File S1: Sequences of all primers designed for this study and relative position according to reference genome KP231180; Supplementary File S2: Different combinations of primers designed to amplify *C. cayetanensis* DNA; Supplementary File S3: Multiple sequence across different members of Eimeriidae homologous to 182 bp fragment from *C. cayetanensis* mitochondria reference sequence. Supplementary File S4: Alignment of a representative subset across closest related members of Eimeriidae homologous to 182 bp fragment from *C. cayetanensis* mitochondria reference sequence from Supplementary File S3.
