# Supplementary material for: Development of a Molecular Marker Based on the Mitochondrial Genome for Detection of Cyclospora cayetanensis in Food and Water Samples
Source: Microorganisms. 2022 Aug 31;10(9):1762. doi: 10.3390/microorganisms10091762 (PMC9504131; doi:10.3390/microorganisms10091762)
Supplement: Supplementary file 1 [file microorganisms-10-01762-s001.zip › Supplementary file S1.pdf]

Supplemental File S1. Sequences of all primers designed for this study and relative position according to reference genome KP231180.

Designation	Sequence (5'–3')	Primer length	TM*	Position
3F1	TGCCAAACTATTCAAACAATCTTCTCA	27	53.7	3832 to 3858
3F2	AATGCCAAACTATTCAAACAATCTTCTC	28	54.1	3830 to 3858
3F3	TGCCGGGCAGATGTCATAAA	20	51.8	3732 to 3751
3F4	GTTTGAATCCAACAGACGCTTTC	23	53.5	3786 to 3808
3R1	CCTTTCCGGTTGTTTCCATCTC	22	54.8	3992 to 4013
3R2	TTTAGGACATGCAGTAACCTTTCCG	25	56	4005 to 4030
3R3	CTAATACAGTGAGCAAGAATGGTGAAAAT	29	56	4339 to 4367
3R4	TAATTAGCGGTTAAATGTCAATCATACA	28	52.6	4101 to 4128
4F1	GTACTACATCAGCTTCTCTGGTTTCA	26	56.4	4398 to 4423
4F2	GGTTTCATCAATTTGTTTAGGTGTTA	26	51.7	4417 to 4442
4F3	TACTACATCAGCTTCTCTGGTTTCAT	26	54.8	4399 to 4424
4R1	CATAAGAATACACATGATGCTCCAG	25	54.4	351 to 375
F3R5	AGAGATACTTACAAGACCCTCAGT	24	54	4541 to 4564
F3F5a	TGATGATTGCTATTCCTACTGG	22	51.1	2179 to 2200
F3R6	TAGAGATACTTACAAGACCCTCAG	24	54	4542 to 4565

*TM = Melting temperature
