# Supplementary material for: Development of a Molecular Marker Based on the Mitochondrial Genome for Detection of Cyclospora cayetanensis in Food and Water Samples
Source: Microorganisms. 2022 Aug 31;10(9):1762. doi: 10.3390/microorganisms10091762 (PMC9504131; doi:10.3390/microorganisms10091762)
Supplement: Supplementary file 1 [file microorganisms-10-01762-s001.zip › Supplementary file S2.pdf]

Supplemental File S2. Different combinations of primers designed to amplify *C. cayetanensis* DNA.

Primer F	Primer R	TM*	Fragment Size (bp)
3F1	3R1	56.8	182
3F1	3R2	57.5	199
3F2	3R1	56.5	184
3F2	3R2	57.1	201
3F1	3R3	57	536
3F2	3R3	56.5	538
3F3	3R4	55.6	397
3F4	3R4	55	343
F3F5a	F3R5	48.1	2386
F3F5a	F3R6	47.6	2387
4F1	4R1	52.8	2252
4F2	4R1	52.7	2233
4F3	4R1	52.6	2251

*TM = Melting temperature
