# Supplementary material for: Development of a Molecular Marker Based on the Mitochondrial Genome for Detection of Cyclospora cayetanensis in Food and Water Samples
Source: Microorganisms. 2022 Aug 31;10(9):1762. doi: 10.3390/microorganisms10091762 (PMC9504131; doi:10.3390/microorganisms10091762)
Supplement: Supplementary file 1 [file microorganisms-10-01762-s001.zip › Supplementary file S3.pdf]

Supplemental File S3. Multiple sequence across different members of Eimeriidae homologous to 182 bp fragment from *C. cayetanensis* mitochondria reference sequence.

>ref_3832_4013

TGCCAAACTATTCAAACAATCTTCTCACTTTCTTATTAATGGAAGCGCTGGTACCTGGGTATCCAATCCA

GTGCTCCTAATTCGGCATAGAGACTCAGCCTCAGTCCAACTTTGTACTGTTTTTTACCAAAAGGGACTCC

ATAAGTTAAACTGTAGAGTCGAGATGGAAACAACCGGAAAGG

>KX495129.1:3835-4016 Eimeria falciformis mitochondrion, complete genome

TGCCAAACTATTCAAACAATATTATAACTTTCTTATTAATGGAAGCGCTGGTACCTGGGTATCCAATCCAGTGCTCC
TCA

TTCGGCATAGAGACTCAGCCTCAGTTCAACTTTGTACTGTTTTTTACCAAAGGGGACTCCAGAAGTTAAACTGTAG
AGTC

GAGATGGAAACAACCGGAAAGG

>KP025690.1:3860-4041 Eimeria irresidua mitochondrion, complete genome

TGCCAAACTATTCAAGCAATTATATCACTTTCTTATTAATGGAAGCGCTGGTACCTGGGTATCCAATCCAGTGCTCC
TCA

TTCGGCATAGAGACTCAGCCTCAGTTCAACTTTGTACTGTTTTTTACCAAAAGGGACTCCAGAAGTTAAACTGTAGA
GTC

GAGATGGAAACAACCGGAAAGG

>KT203397.1:3966-4147 Isospora sp. JRB-2016 mitochondrion, complete genome

TGCCAAACTATTCAAACAATATTATTACTTTCTTATTAATGGAAGCGCTGGTACCTGGGTATCCAATCCAGTGCTCC
TCA

TTCGGCATAGAGACTCAGCCTCAGTTCAACTTTGTACTGTTTTTTACCAAAGAGGACTCCAGAAGTTAAACTGTAGA
GTC

GAGATGGAAACAACCGGAAAGG

>KP025693.1:3860-4041 Eimeria flavescens mitochondrion, complete genome

TGCCAAACTATTCAAGCAATTATATCACTTTCTTATTAATGGAAGCGCTGGTACCTGGGTATCCAATCCAGTGCTCC
TCA

TTCGGCATAGAGACTCAGCCTCTGTTCAACTTTGTACTGTTTTTTACCAAAAGGGACTCCAGAAGTTAAACTGTAGA
GTC

GAGATGGAAACAACCGGAAAGG

>MW303519.1:3968-4149 Isospora lugensae isolate 20236 mitochondrion, complete genome

TGCCAAACTATTCAAACAATATTATTACTTTCTTATTAATGGAAGCGCTGGTACCTGGGTATCCAATCCAGTGCTCC
TCA

TTCGGCATAGAGACTCAGCCTCAGTTCAACTTTGTACTGTTTTTTACCAAAGAGGACTCCAGAAGTTAAACTGTAGA
GTC

GAGATGGAAACAACCGGAAAGG

>MW934557.1:3968-4146 Eimeria legionensis isolate JMR 2018_el mitochondrion, complete genome

TGCCAAACTATTCAAACATTCTTACTTTCTTATTAATGGAAGCGCTGGTACCTGGGTATCCAATCCAGTGCTCCTCAT
TC

GGCATAGAGACTCAGCCTCAGTTCAACATTGTACTGTTTTTTACAAAAAGGGACTCCATAAGTTAAACTGTAGAGT
CGAG

ATGGAAACAACCGGAAAGG

>MW934556.1:3995-4173 Eimeria kofoidi isolate JMR 2018_ek mitochondrion, complete genome

TGCCAAACTATTCAAACATTATTACTTTCTTATTAATGGAAGCGCTGGTACCTGGGTATCCAATCCAGTGCTCCTCAT
TC

GGCATAGAGACTCAGCCTCAGTTCAACTTTGTACTGTTTTTTACAAAAAGGGACTCCATAAGTTAAACTGTAGAGT
CGAG

ATGGAAACAACCGGAAAGG

>KX276861.2:3952-4131 Isospora manorinae isolate 22795 mitochondrion, complete genome

TGCCAAACTATTCAAACAATTATTACTTTCTTATTAATGGAAGCGCTGGTACCTGGGTATCCAATCCAGTGCTCCTC
ATT

CGGCATAGAGACTCAGCCTCAGTCCAACTTTGTACTGTTTTTTACCAAAGAGGACTCCAGAAGTTAAACTGTAGAG
TCGA

GATGGAAACAACCGGAAAGG

>KP009592.1:3850-4031 Eimeria intestinalis mitochondrion, complete genome

TGCCAAACTATTCAAGCAATATTATTACTTTCTTATTAATGGAAGCGCTGGTACCTGGGTATCCAATCCAGTGCTCC
TCA

TTCGGCATAGAGACTCAGCCTCTGTTCAACTTTGTACTGTTTTTTACCAAAAAGGACTCCAGAAGTTAAACTGTAGA
GTC

GAGATGGAAACAACCGGAAAGG

>KP658103.1:3961-4142 Isospora sp. JRB-2015 voucher AHL_G09-038458 mitochondrion, complete genome

TGCCAAACTATTCAAGCAATTTTATTACTTTCTTATTAATGGAAGCGCTGGTACCTGGGTATCCAATCCAGTGCTCCT
CA

TTCGGCATAGAGACTCAGCCTCAGTTCAACTTTGTACTGTTTTTTACCAAAGAGGACTCCAGAAGTTAAACTGTAGA
GTC

GAGATGGAAACAACCGGAAAGG

>KR108296.1:3959-4146 Eimeria innocua mitochondrion, complete genome

TGCCAAACTATTTTTTTTCAAACGATCTTATAACTTTCTTATTAATGGAAGCGCTGGTACCTGGGTATCCAATCCAGT
GC

TCCTCATTCGGCATAGAGACTCAGCCTCAGTTCAACTTTGTACTGTTTTTTACCAAAAGGGACTCCAGAAGTTAAAC
TGT

AGAGTCGAGATGGAAACAACCGGAAAGG

>KJ608416.1:3923-4110 Eimeria dispersa mitochondrion, complete genome

TGCCAAACTATGTTTTTTCAAACTATCTTATAACTTTCTTATTAATGGAAGCGCTGGTACCTGGGTATCCAATCCAGT
GC

TCCTCATTCGGCATAGAGACTCAGCCTCAGTTCAACTTTGTACTGTTTTTTACCAAAAGGGACTCCAGAAGTTAAAC
TGT

AGAGTCGAGATGGAAACAACCGGAAAGG

>MW934554.1:3969-4147 Eimeria chapmani isolate CMNPA 2021-0006 mitochondrion, complete
genome

TGCCAAACTATTCAAACATTATTACTTTCTTATTAATGGAAGCGCTGGTACCTGGGTATCCAATCCAGTACTCCTCAT
TC

GGCATAGAGACTCAGCCTCAGTTCAACTTTGTACTGTTTTTTACAAAAAGGGACTCCATAAGTTAAACTGTAGAGT
CGAG

ATGGAAACAACCGGAAAGG

>KX094951.1:3828-4007 Eimeria tenella strain Ingten mitochondrion, complete genome

TGCCAAACTATTCAAACAATATTACTTTCTTATTAATGGAAGCGCTGGTACCTGGGTATCCAATCCAGTGCTCCTCA
TTC

GGCATAGAGACTCAGCCTCAGTCCAACTTTGTACTGGTTTTTAATAAAAAGGGACTCCATAAGTTAAACTGTAGAG
TCGA

GATGGAAACAACCGGAAAGG

>KX094950.1:3828-4007 Eimeria tenella strain Redten mitochondrion, complete genome

TGCCAAACTATTCAAACAATATTACTTTCTTATTAATGGAAGCGCTGGTACCTGGGTATCCAATCCAGTGCTCCTCA
TTC

GGCATAGAGACTCAGCCTCAGTCCAACTTTGTACTGGTTTTTAATAAAAAGGGACTCCATAAGTTAAACTGTAGAG
TCGA

GATGGAAACAACCGGAAAGG

>KX094949.1:3828-4007 Eimeria tenella strain Darten mitochondrion, complete genome

TGCCAAACTATTCAAACAATATTACTTTCTTATTAATGGAAGCGCTGGTACCTGGGTATCCAATCCAGTGCTCCTCA
TTC

GGCATAGAGACTCAGCCTCAGTCCAACTTTGTACTGGTTTTTAATAAAAAGGGACTCCATAAGTTAAACTGTAGAG
TCGA

GATGGAAACAACCGGAAAGG

>KR108297.1:3969-4149 Isospora amphiboluri voucher AHL12-019929 mitochondrion, complete genome

TGCCAAACTCTTCAAACAATATTTAACTTTCTTATTAATGGAAGCGCTGGTACCTGGGTATCCAATCCAGTGCTCCT
CAT

TCGGCATAGAGACTCAGCCTCTGTTCAACCTTGTACTGTTTTTTACCAAAAGGGACTCCAGAAGTTAAACTGTAGA
GTCG

AGATGGAAACAACCGGAAAGG

>KJ608418.1:3914-4093 Eimeria meleagridis mitochondrion, complete genome

TGCCAAACTATTCAAACAATACTACTTTCTTATTAATGGAAGCGCTGGTACCTGGGTATCCAATCCAGTGCTCCTCA
TTC

GGCATAGAGACTCAGCCTCAGTCCAACTTTGTACTGATTTTTAACAAAAAGGGACTCCATAAGTTAAACTGTAGAG
TCGA

GATGGAAACAACCGGAAAGG

>KJ608413.1:3916-4095 Eimeria gallopavonis mitochondrion, complete genome

TGCCAAACTATTCAAACAATATTACTTTCTTATTAATGGAAGCGCTGGTACCTGGGTATCCAATCCAGTGCTCCTCA
TTC

GGCATAGAGACTCAGCCTCTGTCCAACTTTGTACTGGTTTTTAACAAAAAGGGACTCCATAAGTTAAACTGTAGAG
TCGA

GATGGAAACAACCGGAAAGG

>HG994976.1:3828-4007 Eimeria tenella genome assembly, organelle: mitochondrion

TGCCAAACTATTCAAACAATATTACTTTCTTATTAATGGAAGCGCTGGTACCTGGGTATCCAATCCAGTGCTCCTCA
TTC

GGCATAGAGACTCAGCCTCAGTCCAACTTTGTACTGGTTTTTAATAAAAAGGGACTCCATAAGTTAAACTGTAGAG
TCGA

GATGGAAACAACCGGAAAGG

>HQ702484.1:3828-4007 Eimeria tenella mitochondrion, complete genome

TGCCAAACTATTCAAACAATATTACTTTCTTATTAATGGAAGCGCTGGTACCTGGGTATCCAATCCAGTGCTCCTCA
TTC

GGCATAGAGACTCAGCCTCAGTCCAACTTTGTACTGGTTTTTAATAAAAAGGGACTCCATAAGTTAAACTGTAGAG
TCGA

GATGGAAACAACCGGAAAGG

>AB564272.1:3773-3952 Eimeria tenella concatemeric mitochondrial DNA, unit sequence, strain: NIAH

TGCCAAACTATTCAAACAATATTACTTTCTTATTAATGGAAGCGCTGGTACCTGGGTATCCAATCCAGTGCTCCTCA
TTC

GGCATAGAGACTCAGCCTCAGTCCAACTTTGTACTGGTTTTTAATAAAAAGGGACTCCATAAGTTAAACTGTAGAG
TCGA

GATGGAAACAACCGGAAAGG

>KX495130.1:3817-3998 Eimeria zuernii mitochondrion, complete genome

TGCCAAACTATTCAAACAATTATAGAACTTTCTTATTAATGGAAGCGCTGGTACCTGGGTATCCAATCCAGTGCTCC
TCA

TTCGGCATAGAGACTCAGCCTCAGTTCAACTTTGTACTGTGTTTTACCAAAGGGGACTCCAGAAGTTAAACTGTAG
AGTC

GAGATGGAAACAACCGGAAAGG

>KT203398.1:3922-4103 Eimeria mephitidis strain OVC_2015-05-15 mitochondrion, complete genome

TGCCAAACTCTTCAAACAATGCAATTACTTTCTTATTAATGGAAGCGCTGGTACCTGGGTATCCAATCCAGTGCTCC
TCA

TTCGGCATAGAGACTCAGCCTCAGTTCAACTTTGTACTGTTTTTTACCAAAGGGGACTCCAGAAGTTAAACTGTAG
AGTC

GAGATGGAAACAACCGGAAAGG

>KP025692.1:3841-4022 Eimeria vejdovskyi mitochondrion, complete genome

TGCCAAACTATTCAAGCAATATTATTACTTTCTTATTAATGGAAGCGCTGGTACCTGGGTATCCAATCCAGTGCTCC
TCA

TTCGGCATAGAGACTCAGCCTCTGTTCAACTTTGTACTGTTTTTTACCAAAGAGGACTCCAGAAGTTAAACTGTAGA
GTC

GAGATGGAAACAACCGGAAAGG

>KP025691.1:3846-4027 Eimeria media mitochondrion, complete genome

TGCCAAACTATTCAAGCAATATTATTACTTTCTTATTAATGGAAGCGCTGGTACCTGGGTATCCAATCCAGTGCTCC
TCA

TTCGGCATAGAGACTCAGCCTCTGTTCAACTTTGTACTGTTTTTTACCAAAGAGGACTCCAGAAGTTAAACTGTAGA
GTC

GAGATGGAAACAACCGGAAAGG

>KF419217.1:3847-4028 Eimeria magna mitochondrion, complete genome

TGCCAAACTATTCAAGCAATATTATTACTTTCTTATTAATGGAAGCGCTGGTACCTGGGTATCCAATCCAGTGCTCC
TCA

TTCGGCATAGAGACTCAGCCTCTGTTCAACTTTGTACTGTTTTTTACCAAAGAGGACTCCAGAAGTTAAACTGTAGA
GTC

GAGATGGAAACAACCGGAAAGG

>MW354691.1:3984-4165 Eimeria leuckarti mitochondrion, complete genome

TGCCAAACTATTCAAACAATGTTATAACTTTCTTATTAATGGAAGCGCTGGTACCTGGGTATCCAATCCAGTGCTCC
TCA

TTCGGCATAGAGACTCAGCCTATGTTCAACTTTGTACTGTTTTTTACCAAAGAGGACTCCATAAGTTAAACTGTAGA
GTC

AAGATGGAAACAACCGGAAAGG

>KX094948.1:3831-4009 Eimeria acervulina strain Ponace mitochondrion, complete genome

TGCCAAACTATTCAAACATTAGTACTTTCTTATTAATGGAAGCGCTGGTACCTGGGTATCCAATCCAGTGCTCCTCA
TTC

GGCATAGAGACTCAGCCTCAGTCCAACTTTGTACTGATTTTTATAAAAAGGGACTCCATAAGTTAAACTGTAGAGT
CGAG

ATGGAAACAACCGGAAAGG

>KX094947.1:3831-4009 Eimeria acervulina strain Royace mitochondrion, complete genome

TGCCAAACTATTCAAACATTAGTACTTTCTTATTAATGGAAGCGCTGGTACCTGGGTATCCAATCCAGTGCTCCTCA
TTC

GGCATAGAGACTCAGCCTCAGTCCAACTTTGTACTGATTTTTATAAAAAGGGACTCCATAAGTTAAACTGTAGAGT
CGAG

ATGGAAACAACCGGAAAGG

>KX094946.1:3831-4009 Eimeria acervulina strain Newace mitochondrion, complete genome

TGCCAAACTATTCAAACATTAGTACTTTCTTATTAATGGAAGCGCTGGTACCTGGGTATCCAATCCAGTGCTCCTCA
TTC

GGCATAGAGACTCAGCCTCAGTCCAACTTTGTACTGATTTTTATAAAAAGGGACTCCATAAGTTAAACTGTAGAGT
CGAG

ATGGAAACAACCGGAAAGG

>HQ702479.1:3831-4009 Eimeria acervulina mitochondrion, complete genome

TGCCAAACTATTCAAACATTAGTACTTTCTTATTAATGGAAGCGCTGGTACCTGGGTATCCAATCCAGTGCTCCTCA
TTC

GGCATAGAGACTCAGCCTCAGTCCAACTTTGTACTGATTTTTATAAAAAGGGACTCCATAAGTTAAACTGTAGAGT
CGAG

ATGGAAACAACCGGAAAGG

>KX094954.1:3828-4007 Eimeria necatrix strain Gatnec mitochondrion, complete genome

TGCCAAACTATTCAAATAATATTACTTTCTTATTAATGGAAGCGCTGGTACCTGGGTATCCAATCCAGTGCTCCTCA
TTC

GGCATAGAGACTCAGCCTCAGTCCAACTTTGTACTGGTTTTTAATAAAAAGGGACTCCATAAGTTAAACTGTAGAG
TCGA

GATGGAAACAACCGGAAAGG

>KX094953.1:3828-4007 Eimeria necatrix strain Mednec mitochondrion, complete genome

TGCCAAACTATTCAAATAATATTACTTTCTTATTAATGGAAGCGCTGGTACCTGGGTATCCAATCCAGTGCTCCTCA
TTC

GGCATAGAGACTCAGCCTCAGTCCAACTTTGTACTGGTTTTTAATAAAAAGGGACTCCATAAGTTAAACTGTAGAG
TCGA

GATGGAAACAACCGGAAAGG

>KX094952.1:3828-4007 Eimeria necatrix strain Gronec mitochondrion, complete genome

TGCCAAACTATTCAAATAATATTACTTTCTTATTAATGGAAGCGCTGGTACCTGGGTATCCAATCCAGTGCTCCTCA
TTC

GGCATAGAGACTCAGCCTCAGTCCAACTTTGTACTGGTTTTTAATAAAAAGGGACTCCATAAGTTAAACTGTAGAG
TCGA

GATGGAAACAACCGGAAAGG

>KJ608415.1:3914-4093 Eimeria adenoeides mitochondrion, complete genome

TGCCAAACTATTCAAACAATACTACTTTCTTATTAATGGAAGCGCTGGTACCTGGGTATCCAATCCAGTGCTCCTCA
TTC

GGCATAGAGACTCAGCCTCAGTCCAACTTTGTACTGGTTTTTAATAAAAAGGGACTCCATAAGTTAAACTGTAGAG
TCGA

GATGGAAACAACCGGAAAGG

>KR108298.1:3961-4142 Isospora greineri voucher MAH-2013a_MTZ1 mitochondrion, complete genome

TGCCAAACTATTCAAGCAACTATRTTACTTTCTTATTAATGGAAGCGCTGGTACCTGGGTATCCAATCCAGTGCTCC TCA

TTCGGCATAGAGACTCAGCCTCAGTTCAACTTTGTACTGTTTTTTACCAAAGAGGACTCCAGAAGTTAAACTGTAGA GTC

GAGATGGAAACAACCGGAAAGG

>KT203396.1:3961-4142 Isospora superbusi strain MTZ2 mitochondrion, complete genome

TGCCAAACTATTCAAGCAACTATRTTACTTTCTTATTAATGGAAGCGCTGGTACCTGGGTATCCAATCCAGTGCTCC TCA

TTCGGCATAGAGACTCAGCCTCAGTTCAACTTTGTACTGTTTTTTACCAAAGAGGACTCCAGAAGTTAAACTGTAGA GTC

GAGATGGAAACAACCGGAAAGG

>KX276860.3:3974-4155 Isospora serinuse isolate 20237Canary mitochondrion, complete genome

TGCCAAACTGTTCAAACAATATTATTACTTTCTTATTAATGGAAGCGCTGGTACCTGGGTATCCAATCCAGTGCTCC TCA

TTCGGCATAGAGACTCAGCCTCAGTTCAACCTTGTACTGTTTTTTACCAAAGAGGACTCTAGAAGTTAAACTGTAGA GTC

GAGATGGAAACAACCGGAAAGG

>KP658102.1:3997-4178 Caryospora bigenetica voucher C_bigenetica_MTZ_07-12108 mitochondrion, complete genome

TGCCAAACTCTTCAAACAATTCTTAAACTTTCTTATTAATGGAAGCGCTGGTACCTGGGTATCCAATCCAGTGCTCC TCA

TTCGGCATAGAGACTCAGCCTCTGTTCAACCTTGTACTGTTTTTTACCAAAAGGGACTCCAGAAGTTAAACTGTAGA GTC

GAGATGGAAACAACCGGAAAGG

>KX094956.1:3828-4006 Eimeria sp. strain OTU-Z2 mitochondrion, complete genome

TGCCAAACTATTCAAACATTATTACTTTCTTATTAATGGAAGCGCTGGTACCTGGGTATCCAATCCAGTGCTCCTCAT TC

GGCATAGAGACTCAGCCTCTGTCCAACTTTGTACTGATTTTTATAAAAAAGGACTCCATAAGTTAAACTGTAGAGTC GAG

ATGGAAACAACCGGAAAGG

>KX094955.1:3828-4006 Eimeria sp. strain OTU-Z1 mitochondrion, complete genome

TGCCAAACTATTCAAACATTATTACTTTCTTATTAATGGAAGCGCTGGTACCTGGGTATCCAATCCAGTGCTCCTCAT
TC

GGCATAGAGACTCAGCCTCTGTCCAACTTTGTACTGATTTTTATAAAAAAGGACTCCATAAGTTAAACTGTAGAGTC
GAG

ATGGAAACAACCGGAAAGG

>KX094945.1:3825-4003 Eimeria praecox strain Ingpra mitochondrion, complete genome

TGCCAAACTATTCAAACATTTATACTTTCTTATTAATGGAAGCGCTGGTACCTGGGTTTCCAATCCAGTGCTCCTCAT
TC

GGCATAGAGACTCAGCCTCAGTCCAACTTTGTACTGATTTTTATAAAAAGGGACTCCATAAGTTAAACTGTAGAGT
CGAG

ATGGAAACAACCGGAAAGG

>KX094944.1:3825-4003 Eimeria praecox strain Jorpra mitochondrion, complete genome

TGCCAAACTATTCAAACATTTATACTTTCTTATTAATGGAAGCGCTGGTACCTGGGTTTCCAATCCAGTGCTCCTCAT
TC

GGCATAGAGACTCAGCCTCAGTCCAACTTTGTACTGATTTTTATAAAAAGGGACTCCATAAGTTAAACTGTAGAGT
CGAG

ATGGAAACAACCGGAAAGG

>KX094943.1:3825-4003 Eimeria praecox strain Andpra mitochondrion, complete genome

TGCCAAACTATTCAAACATTTATACTTTCTTATTAATGGAAGCGCTGGTACCTGGGTTTCCAATCCAGTGCTCCTCAT
TC

GGCATAGAGACTCAGCCTCAGTCCAACTTTGTACTGATTTTTATAAAAAGGGACTCCATAAGTTAAACTGTAGAGT
CGAG

ATGGAAACAACCGGAAAGG

>HQ702483.1:3826-4004 Eimeria praecox mitochondrion, complete genome

TGCCAAACTATTCAAACATTTATACTTTCTTATTAATGGAAGCGCTGGTACCTGGGTTTCCAATCCAGTGCTCCTCAT
TC

GGCATAGAGACTCAGCCTCAGTCCAACTTTGTACTGATTTTTATAAAAAGGGACTCCATAAGTTAAACTGTAGAGT
CGAG

ATGGAAACAACCGGAAAGG

>MW934555.1:3942-4121 Eimeria sp. JRBarta-2021b isolate OVC 2018 mitochondrion, complete
genome

TGCCAAACTATTCAAACATGAAGACTTTCTTATTAATGGAAGCGCTGGTACCTGGGTATCCAATCCAGTGCTCCTCA
TTC

GGCATAGAGACTCAGCCTCTGTCCAACTTTGTACTGTTTTTTAACAAAAAGGGACTCCATAAGTTAAACTGTAGAG
TCGA

GATGGAAACAACCGGAAAGG

>KX094963.1:3897-4075 Eimeria mitis strain Jormit mitochondrion, complete genome

TGCCAAATTATTCAAACATTAGGACTTTCTTATTAATGGAAGCGCTGGTACCTGGGTATCCAATCCAGTGCTCCTCA
TTC

GGCATAGAGACTCAGCCTCAGTCCAACTTTGTACTGATTTTTATAAAAATGGACTCCATAAGTTAAACTGTAGAGTC
GAG

ATGGAAACAACCGGAAAGG

>KX094962.1:3896-4074 Eimeria mitis strain Redmit mitochondrion, complete genome

TGCCAAATTATTCAAACATTAGGACTTTCTTATTAATGGAAGCGCTGGTACCTGGGTATCCAATCCAGTGCTCCTCA
TTC

GGCATAGAGACTCAGCCTCAGTCCAACTTTGTACTGATTTTTATAAAAATGGACTCCATAAGTTAAACTGTAGAGTC
GAG

ATGGAAACAACCGGAAAGG

>KX094961.1:3896-4074 Eimeria mitis strain Kelmit mitochondrion, complete genome

TGCCAAATTATTCAAACATTAGGACTTTCTTATTAATGGAAGCGCTGGTACCTGGGTATCCAATCCAGTGCTCCTCA
TTC

GGCATAGAGACTCAGCCTCAGTCCAACTTTGTACTGATTTTTATAAAAATGGACTCCATAAGTTAAACTGTAGAGTC
GAG

ATGGAAACAACCGGAAAGG

>KC409031.1:3896-4074 Eimeria mitis clone 24 mitochondrion, complete genome

TGCCAAATTATTCAAACATTAGGACTTTCTTATTAATGGAAGCGCTGGTACCTGGGTATCCAATCCAGTGCTCCTCA
TTC

GGCATAGAGACTCAGCCTCAGTCCAACTTTGTACTGATTTTTATAAAAATGGACTCCATAAGTTAAACTGTAGAGTC
GAG

ATGGAAACAACCGGAAAGG

>KC409030.1:3896-4074 Eimeria mitis clone 20 mitochondrion, complete genome

TGCCAAATTATTCAAACATTAGGACTTTCTTATTAATGGAAGCGCTGGTACCTGGGTATCCAATCCAGTGCTCCTCA
TTC

GGCATAGAGACTCAGCCTCAGTCCAACTTTGTACTGATTTTTATAAAAATGGACTCCATAAGTTAAACTGTAGAGTC
GAG

ATGGAAACAACCGGAAAGG

>KC409029.1:3895-4073 Eimeria mitis clone 18 mitochondrion, complete genome

TGCCAAATTATTCAAACATTAGGACTTTCTTATTAATGGAAGCGCTGGTACCTGGGTATCCAATCCAGTGCTCCTCATTC

GGCATAGAGACTCAGCCTCAGTCCAACTTTGTACTGATTTTTATAAAAATGGACTCCATAAGTTAAACTGTAGAGTCGAG

ATGGAAACAACCGGAAAGG

>KF501573.1:3896-4074 Eimeria mitis mitochondrion, complete genome

TGCCAAATTATTCAAACATTAGGACTTTCTTATTAATGGAAGCGCTGGTACCTGGGTATCCAATCCAGTGCTCCTCATTC

GGCATAGAGACTCAGCCTCAGTCCAACTTTGTACTGATTTTTATAAAAATGGACTCCATAAGTTAAACTGTAGAGTCGAG

ATGGAAACAACCGGAAAGG

>JN864949.1:3896-4074 Eimeria mitis mitochondrion, complete genome

TGCCAAATTATTCAAACATTAGGACTTTCTTATTAATGGAAGCGCTGGTACCTGGGTATCCAATCCAGTGCTCCTCATTC

GGCATAGAGACTCAGCCTCAGTCCAACTTTGTACTGATTTTTATAAAAATGGACTCCATAAGTTAAACTGTAGAGTCGAG

ATGGAAACAACCGGAAAGG

>HQ702482.1:3830-4008 Eimeria necatrix mitochondrion, complete genome

TGCCAAACTATTAAAAAATAATACTTTCTTATTAATGGAAGCGCTGGTACCTGGGTATCCAATCCAGTGCTCCTCATTCG

GCATAGAGACTCAGCCTCAGTCCAACTTTGTACTGGTTTTTAATAAAAAGGGACTCCATAAGTTAAACTGTAGAGTCGAG

ATGGAAACAACCGGAAAGG

>NC_039745.1:3921-4102 Eimeria furonis voucher EU-9014 mitochondrion, complete genome

TGCCAAACTCATCAAACAATACTATTACTTTCTTATTAATGGAAGCGCTGGTGCCTGGGTATCCAATCCAGTACTCCTCA

TTCGGCATAGAGACTCTGCCTCTGTTCAACATTGTACTGTTTTTTACCAAAGGGGACTCCATAAGTTAAACTGTAGAGTC

GAGATGGAAACAACCGGAAAGG

>MF795598.1:3921-4102 Eimeria furonis voucher EU-9014 mitochondrion, complete genome

TGCCAAACTCATCAAACAATACTATTACTTTCTTATTAATGGAAGCGCTGGTGCCTGGGTATCCAATCCAGTACTCCTCA

TTCGGCATAGAGACTCTGCCTCTGTTCAACATTGTACTGTTTTTTACCAAAGGGGACTCCATAAGTTAAACTGTAGA
GTC

GAGATGGAAACAACCGGAAAGG

>KT203399.1:3920-4101 Eimeria cf. ictidea JRB-2016 strain MTZ_BFF_2014 mitochondrion, complete genome

TGCCAAACTCATCAAACAATGTTAGTACTTTCTTATTAATGGAAGCGCTGGTGCCTGGGTATCCAATCCAGTACTCC
TCA

TTCGGCATAGAGACTCTGCCTCTGTTCAACATTGTACTGTTTTTTACCAAAGGGGACTCCATAAGTTAAACTGTAGA
GTC

GAGATGGAAACAACCGGAAAGG

>KJ608414.1:3903-4082 Eimeria meleagrimitis mitochondrion, complete genome

TGCCAAACTATTCAAATATTATTACTTTCTTATTAATGGAAGCGCTGGTACCTGGGTATCCAATCCAGTGCTCCTCAC
TC

GGCATAGAGACTCAGCCTCTGTCCAACTTTGTACTGGATTTTAACAAAAAGGGACTCCACAAGTTAAACTGTAGAG
TCGA

GATGGAAACAACCGGAAAGG

>MH758793.1:3831-4009 Eimeria anseris mitochondrion, complete genome

TGCCAAACTATTCAAACATTAGTACTTTCTTATTAATGGAAGCGCTGGTACCTGGGTATCCAATCCAGTGCTCCTCA
TTC

GGCATAGAGACTCAGCCTCAGTCCAACTTTGTACTGATTTTTATATAAAGGGAATCCATATGTTAAACTGAAGAGT
CGAG

ATGGAAACAACCGGATAGG

>KX094959.1:3820-3998 Eimeria brunetti strain Roybru mitochondrion, complete genome

TGCCAAACTATTCAAACGCTATTACTTTCTTATTAATGGAAGCGCTGGTACCTGGGTATCCAATCCAGTGCTCCTCA
TTC

GGCATAGAGACTCAGCCTCAGTCCAACTTTGTACTGATTTTTATAAAAAAGGACTATACAAGTTAAACTGTATAGTC
GAG

ATGGAAACAACCGGAAAGG

>KX094958.1:3820-3998 Eimeria brunetti strain Monbru mitochondrion, complete genome

TGCCAAACTATTCAAACGCTATTACTTTCTTATTAATGGAAGCGCTGGTACCTGGGTATCCAATCCAGTGCTCCTCA
TTC

GGCATAGAGACTCAGCCTCAGTCCAACTTTGTACTGATTTTTATAAAAAAGGACTATACAAGTTAAACTGTATAGTC
GAG

ATGGAAACAACCGGAAAGG

>KX094957.1:3820-3998 Eimeria brunetti strain Bowbru mitochondrion, complete genome

TGCCAAACTATTCAAACGCTATTACTTTCTTATTAATGGAAGCGCTGGTACCTGGGTATCCAATCCAGTGCTCCTCA
TTC

GGCATAGAGACTCAGCCTCAGTCCAACTTTGTACTGATTTTTATAAAAAAGGACTATACAAGTTAAACTGTATAGTC
GAG

ATGGAAACAACCGGAAAGG

>HQ702480.1:3812-3990 Eimeria brunetti mitochondrion, complete genome

TGCCAAACTATTCAAACGCTATTACTTTCTTATTAATGGAAGCGCTGGTACCTGGGTATCCAATCCAGTGCTCCTCA
TTC

GGCATAGAGACTCAGCCTCAGTCCAACTTTGTACTGATTTTTATAAAAAAGGACTATACAAGTTAAACTGTATAGTC
GAG

ATGGAAACAACCGGAAAGG

>KX094967.1:3813-3991 Eimeria sp. strain OTU-X1 mitochondrion, complete genome

TGCCAAACTATTCAACCAGTAGTACTTTCTTATTAATGGAAGCGCTGGTACCTGGGTATCCAATCCAGTGCTCCTCA
TTC

GGCATAGAGACTCAGCCTCTGTCCAACTTTGTACTGATTTTTATAAAAATGGACTCCACAAGTTAAACTGTTGAGTC
AAG

ATGGAAACAACCGGAAAGG

>KX094966.1:3814-3992 Eimeria maxima strain Ingmax mitochondrion, complete genome

TGCCAAACTATTCAACCATTAGTACTTTCTTATTAATGGAAGCGCTGGTACCTGGGTATCCAATCCAGTGCTCCTCA
TTC

GGCATAGAGACTCAGCCTATGTCCAACTTTGTACTGATTTTTATAAAAATGGACTCCACAAGTTAAACTGTTGAGTC
GAG

ATGGAAACAACCGGAAAGG

>KX094965.1:3814-3992 Eimeria maxima strain Medmax mitochondrion, complete genome

TGCCAAACTATTCAACCATTAGTACTTTCTTATTAATGGAAGCGCTGGTACCTGGGTATCCAATCCAGTGCTCCTCA
TTC

GGCATAGAGACTCAGCCTATGTCCAACTTTGTACTGATTTTTATAAAAATGGACTCCACAAGTTAAACTGTTGAGTC
GAG

ATGGAAACAACCGGAAAGG

>KX094964.1:3814-3992 Eimeria maxima strain ARI-M3 mitochondrion, complete genome

TGCCAAACTATTCAACCATTAGTACTTTCTTATTAATGGAAGCGCTGGTACCTGGGTATCCAATCCAGTGCTCCTCA
TTC

GGCATAGAGACTCAGCCTATGTCCAACTTTGTACTGATTTTTATAAAAATGGACTCCACAAGTTAAACTGTTGAGTC
GAG

ATGGAAACAACCGGAAAGG

>HQ702481.1:3814-3992 Eimeria maxima mitochondrion, complete genome

TGCCAAACTATTCAACCATTAGTACTTTCTTATTAATGGAAGCGCTGGTACCTGGGTATCCAATCCAGTGCTCCTCA
TTC

GGCATAGAGACTCAGCCTATGTCCAACTTTGTACTGATTTTTATAAAAATGGACTCCACAAGTTAAACTGTTGAGTC
GAG

ATGGAAACAACCGGAAAGG

>KX094960.1:3834-4012 Eimeria sp. strain OTU-Y1 mitochondrion, complete genome

TGCCAAATTATTCAAACATTATTCCTTTCTTATTAATGGAAGCGCTGGTACCTGGGTATCCAATCCAGTGCTCCTCAT
TC

GGCATAGAGACTCAGCCTCAGTCCAATCTTGTACTGATTTTTATAAAAAAGGACTATACAAGTTAAACTGTATAGTC
GAG

ATGGAAACAACCGGAAAGG

>KT369006.1:3961-4142 Lankesterella sp. Guelph2013 isolate B mitochondrion, complete genome

TGCCAAACTCTTCAAGCTATATTTATACTTTCTTATTAATGGAAGCGCTGATACCTGGGTATCCAATCCAGTGCTCCT
CA

TTCGGCATAGAGACTCAGCCTCTGTTCAACCTTGTACTGCTTTTTACAAAAATGGACTCCAGAAGTTAACCTGTAGA
GTC

GAGATGGAATCAACCGGAAAGG

>KT369005.1:3961-4142 Lankesterella sp. Guelph2013 isolate A mitochondrion, complete genome

TGCCAAACTCTTCAAGCTATATTGATACTTTCTTATTAATGGAAGCGCTGATACCTGGGTATCCAATCCAGTGCTCC
TCA

TTCGGCATAGAGACTCAGCCTCTGTTCAACCTTGTACTGCTTTTTACAAAAATGGACTCCAGAAGTTAACCTGTAGA
GTC

GAGATGGAATCAACCGGAAAGG

>KT203395.2:3982-4157 Choleoeimeria sp. JRB-2016 mitochondrion, complete genome

TGCCAAACTCTTCAAGAGATACTTTCTTATTGATGGAAGCGCTGGTACCTGGGTATCCAATCCAGTGCTCCTCTCTC
GGC

ATTGAGACTCAGCCTCAGTTCAACCTTGTACTAATTTTTATAAAAAGGGACTCCAGAAGTTAAACTGTTGAGTCAAGATG

GAAACAACCGGAAAGG

>MK813349.1:3959-4134 Choleoeimeria taggarti voucher QM:G466192 mitochondrion, complete genome

TGCCAAACTCTTCAAGAGATACTTTCTTATTGATGGAAGCGCTGGTACCTGGGTATCCAATCCAGTGCTCCTCTCTCGGC

ATTGAGACTCAGCCTTAGTTCAACCTTGAACTATTTTTTATAAAAATGGACTCCAGAAGTTAAACTGTTGAGTCGAGATG

GAAACAACCGGAAAGG
