# Supplementary material for: Development of a Molecular Marker Based on the Mitochondrial Genome for Detection of Cyclospora cayetanensis in Food and Water Samples
Source: Microorganisms. 2022 Aug 31;10(9):1762. doi: 10.3390/microorganisms10091762 (PMC9504131; doi:10.3390/microorganisms10091762)
Supplement: Supplementary file 1 [file microorganisms-10-01762-s001.zip › Supplementary file S4.pdf]

Supplementary File S4. Alignment of a representative subset across closest related members of Eimeriidae homologous to 182 bp fragment from *C. cayetanensis* mitochondria reference sequence from Supplementary File S3.

```
CLUSTAL format alignment by MAFFT (v7.487)


ref_3832_4013   TGCCAAACTA------TTCAAACAATCTTCTCACTTTCTTATTAATGGAAGCGCTGGTAC
E.falciformis   TGCCAAACTA------TTCAAACAATATTATAACTTTCTTATTAATGGAAGCGCTGGTAC
E.intestinalis  TGCCAAACTA------TTCAAGCAATATTATTACTTTCTTATTAATGGAAGCGCTGGTAC
E.innocua       TGCCAAACTATTTTTTTTCAAACGATCTTATAACTTTCTTATTAATGGAAGCGCTGGTAC
E.dispersa      TGCCAAACTATGTTTTTTCAAACTATCTTATAACTTTCTTATTAATGGAAGCGCTGGTAC
E.tenella       TGCCAAACTA------TTCAAACAA---TATTACTTTCTTATTAATGGAAGCGCTGGTAC
E.magna         TGCCAAACTA------TTCAAGCAATATTATTACTTTCTTATTAATGGAAGCGCTGGTAC
E.acervulina    TGCCAAACTA------TTCAAACAT---TAGTACTTTCTTATTAATGGAAGCGCTGGTAC
E.flavescens    TGCCAAACTA------TTCAAGCAATTATATCACTTTCTTATTAATGGAAGCGCTGGTAC
E.sp.OTU-Z2     TGCCAAACTA------TTCAAACAT---TATTACTTTCTTATTAATGGAAGCGCTGGTAC
E.sp.OTU-Z1     TGCCAAACTA------TTCAAACAT---TATTACTTTCTTATTAATGGAAGCGCTGGTAC
E.praecox       TGCCAAACTA------TTCAAACAT---TTATACTTTCTTATTAATGGAAGCGCTGGTAC
E.mitis         TGCCAAATTA------TTCAAACAT---TAGGACTTTCTTATTAATGGAAGCGCTGGTAC
E.necatrix      TGCCAAACTA------TT-AAAAAA---TAATACTTTCTTATTAATGGAAGCGCTGGTAC
E.maxima        TGCCAAACTA------TTCAACCAT---TAGTACTTTCTTATTAATGGAAGCGCTGGTAC
I.lugensae      TGCCAAACTA------TTCAAACAATATTATTACTTTCTTATTAATGGAAGCGCTGGTAC
I.manorinae     TGCCAAACTA------TTCAAACAAT--TATTACTTTCTTATTAATGGAAGCGCTGGTAC
I.sp.           TGCCAAACTA------TTCAAGCAATTTTATTACTTTCTTATTAATGGAAGCGCTGGTAC
I.amphiboluri   TGCCAAACTC------TTCAAACAATA-TTTAACTTTCTTATTAATGGAAGCGCTGGTAC
I.greineri      TGCCAAACTA------TTCAAGCAACTATRTTACTTTCTTATTAATGGAAGCGCTGGTAC
I.superbusi     TGCCAAACTA------TTCAAGCAACTATRTTACTTTCTTATTAATGGAAGCGCTGGTAC
I.serinuse      TGCCAAACTG------TTCAAACAATATTATTACTTTCTTATTAATGGAAGCGCTGGTAC
Lankesterella   TGCCAAACTC------TTCAAGCTATATTTATACTTTCTTATTAATGGAAGCGCTGATAC
                *******.*       ** **       *   ************************.***

ref_3832_4013   CTGGGTATCCAATCCAGTGCTCCTAATTCGGCATAGAGACTCAGCCTCAGTCCAACTTTG
E.falciformis   CTGGGTATCCAATCCAGTGCTCCTCATTCGGCATAGAGACTCAGCCTCAGTTCAACTTTG
E.intestinalis  CTGGGTATCCAATCCAGTGCTCCTCATTCGGCATAGAGACTCAGCCTCTGTTCAACTTTG
E.innocua       CTGGGTATCCAATCCAGTGCTCCTCATTCGGCATAGAGACTCAGCCTCAGTTCAACTTTG
E.dispersa      CTGGGTATCCAATCCAGTGCTCCTCATTCGGCATAGAGACTCAGCCTCAGTTCAACTTTG
E.tenella       CTGGGTATCCAATCCAGTGCTCCTCATTCGGCATAGAGACTCAGCCTCAGTCCAACTTTG
E.magna         CTGGGTATCCAATCCAGTGCTCCTCATTCGGCATAGAGACTCAGCCTCTGTTCAACTTTG
E.acervulina    CTGGGTATCCAATCCAGTGCTCCTCATTCGGCATAGAGACTCAGCCTCAGTCCAACTTTG
E.flavescens    CTGGGTATCCAATCCAGTGCTCCTCATTCGGCATAGAGACTCAGCCTCTGTTCAACTTTG
E.sp.OTU-Z2     CTGGGTATCCAATCCAGTGCTCCTCATTCGGCATAGAGACTCAGCCTCTGTCCAACTTTG
E.sp.OTU-Z1     CTGGGTATCCAATCCAGTGCTCCTCATTCGGCATAGAGACTCAGCCTCTGTCCAACTTTG
E.praecox       CTGGGTTTCCAATCCAGTGCTCCTCATTCGGCATAGAGACTCAGCCTCAGTCCAACTTTG
E.mitis         CTGGGTATCCAATCCAGTGCTCCTCATTCGGCATAGAGACTCAGCCTCAGTCCAACTTTG
E.necatrix      CTGGGTATCCAATCCAGTGCTCCTCATTCGGCATAGAGACTCAGCCTCAGTCCAACTTTG
E.maxima        CTGGGTATCCAATCCAGTGCTCCTCATTCGGCATAGAGACTCAGCCTATGTCCAACTTTG
I.lugensae      CTGGGTATCCAATCCAGTGCTCCTCATTCGGCATAGAGACTCAGCCTCAGTTCAACTTTG
I.manorinae     CTGGGTATCCAATCCAGTGCTCCTCATTCGGCATAGAGACTCAGCCTCAGTCCAACTTTG
I.sp.           CTGGGTATCCAATCCAGTGCTCCTCATTCGGCATAGAGACTCAGCCTCAGTTCAACTTTG
I.amphiboluri   CTGGGTATCCAATCCAGTGCTCCTCATTCGGCATAGAGACTCAGCCTCTGTTCAACCTTG
I.greineri      CTGGGTATCCAATCCAGTGCTCCTCATTCGGCATAGAGACTCAGCCTCAGTTCAACTTTG
I.superbusi     CTGGGTATCCAATCCAGTGCTCCTCATTCGGCATAGAGACTCAGCCTCAGTTCAACTTTG
I.serinuse      CTGGGTATCCAATCCAGTGCTCCTCATTCGGCATAGAGACTCAGCCTCAGTTCAACCTTG
Lankesterella   CTGGGTATCCAATCCAGTGCTCCTCATTCGGCATAGAGACTCAGCCTCTGTTCAACCTTG
                ****** ***************** **********************  **.****.***
```

```
ref_3832_4013   TACTGTTTTTT-ACCAAAAGGGACTCCATAAGTTAAACTGTAGAGTCGAGATGGAAACAA
E.falciformis   TACTGTTTTTT-ACCAAAGGGGACTCCAGAAGTTAAACTGTAGAGTCGAGATGGAAACAA
E.intestinalis  TACTGTTTTTT-ACCAAAAAGGACTCCAGAAGTTAAACTGTAGAGTCGAGATGGAAACAA
E.innocua       TACTGTTTTTT-ACCAAAAGGGACTCCAGAAGTTAAACTGTAGAGTCGAGATGGAAACAA
E.dispersa      TACTGTTTTTT-ACCAAAAGGGACTCCAGAAGTTAAACTGTAGAGTCGAGATGGAAACAA
E.tenella       TACTGGTTTTTAATAAAAAGGGACTCCATAAGTTAAACTGTAGAGTCGAGATGGAAACAA
E.magna         TACTGTTTTTT-ACCAAAGAGGACTCCAGAAGTTAAACTGTAGAGTCGAGATGGAAACAA
E.acervulina    TACTGATTTTT-ATAAAAAGGGACTCCATAAGTTAAACTGTAGAGTCGAGATGGAAACAA
E.flavescens    TACTGTTTTTT-ACCAAAAGGGACTCCAGAAGTTAAACTGTAGAGTCGAGATGGAAACAA
E.sp.OTU-Z2     TACTGATTTTT-ATAAAAAAGGACTCCATAAGTTAAACTGTAGAGTCGAGATGGAAACAA
E.sp.OTU-Z1     TACTGATTTTT-ATAAAAAAGGACTCCATAAGTTAAACTGTAGAGTCGAGATGGAAACAA
E.praecox       TACTGATTTTT-ATAAAAAGGGACTCCATAAGTTAAACTGTAGAGTCGAGATGGAAACAA
E.mitis         TACTGATTTTT-ATAAAAATGGACTCCATAAGTTAAACTGTAGAGTCGAGATGGAAACAA
E.necatrix      TACTGGTTTTTAATAAAAAGGGACTCCATAAGTTAAACTGTAGAGTCGAGATGGAAACAA
E.maxima        TACTGATTTTT-ATAAAAATGGACTCCACAAGTTAAACTGTTGAGTCGAGATGGAAACAA
I.lugensae      TACTGTTTTTT-ACCAAAGAGGACTCCAGAAGTTAAACTGTAGAGTCGAGATGGAAACAA
I.manorinae     TACTGTTTTTT-ACCAAAGAGGACTCCAGAAGTTAAACTGTAGAGTCGAGATGGAAACAA
I.sp.           TACTGTTTTTT-ACCAAAGAGGACTCCAGAAGTTAAACTGTAGAGTCGAGATGGAAACAA
I.amphiboluri   TACTGTTTTTT-ACCAAAAGGGACTCCAGAAGTTAAACTGTAGAGTCGAGATGGAAACAA
I.greineri      TACTGTTTTTT-ACCAAAGAGGACTCCAGAAGTTAAACTGTAGAGTCGAGATGGAAACAA
I.superbusi     TACTGTTTTTT-ACCAAAGAGGACTCCAGAAGTTAAACTGTAGAGTCGAGATGGAAACAA
I.serinuse      TACTGTTTTTT-ACCAAAGAGGACTCTAGAAGTTAAACTGTAGAGTCGAGATGGAAACAA
Lankesterella   TACTGCTTTTT-ACAAAAATGGACTCCAGAAGTTAACCTGTAGAGTCGAGATGGAATCAA
                ***** ***** *. ***. ******.* ******* **** ************** ***

ref_3832_4013   CCGGAAAGG
E.falciformis   CCGGAAAGG
E.intestinalis  CCGGAAAGG
E.innocua       CCGGAAAGG
E.dispersa      CCGGAAAGG
E.tenella       CCGGAAAGG
E.magna         CCGGAAAGG
E.acervulina    CCGGAAAGG
E.flavescens    CCGGAAAGG
E.sp.OTU-Z2     CCGGAAAGG
E.sp.OTU-Z1     CCGGAAAGG
E.praecox       CCGGAAAGG
E.mitis         CCGGAAAGG
E.necatrix      CCGGAAAGG
E.maxima        CCGGAAAGG
I.lugensae      CCGGAAAGG
I.manorinae     CCGGAAAGG
I.sp.           CCGGAAAGG
I.amphiboluri   CCGGAAAGG
I.greineri      CCGGAAAGG
I.superbusi     CCGGAAAGG
I.serinuse      CCGGAAAGG
Lankesterella   CCGGAAAGG
                *********
```
